# Supplementary material for: Bioinformatics Analysis and Expression Profiling Under Abiotic Stress of the DREB Gene Family in Glycyrrhiza uralensis
Source: Int J Mol Sci. 2025 Sep 22;26(18):9235. doi: 10.3390/ijms26189235 (PMC12470958; doi:10.3390/ijms26189235)
Supplement: Supplementary file 1 [file ijms-26-09235-s001.zip › ijms-3812617-Supplementary Materials.pdf]

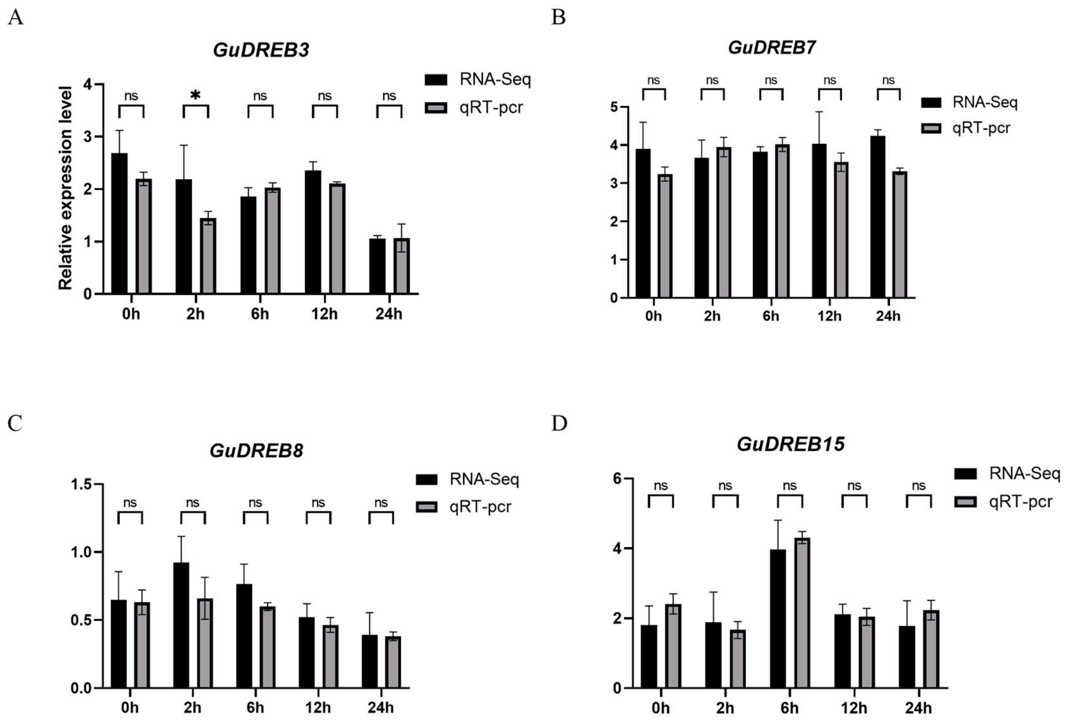


Figure S1. Validation of RNA-seq was performed using qPCR.

Note: * indicates a statistically significant difference ($p < 0.05$) between RNA-Seq and qRT-PCR expression values; ns indicates a non-significant difference.
